# Light People | Prof. Wei Lu spoke about infrared physics

**DOI:** 10.1038/s41377-025-02012-8

**Published:** 2025-09-19

**Authors:** Chenzi Guo, Peng Wang

**Affiliations:** 1 Changchun Institute of Optics, Fine Mechanics and Physics, Chinese Academy of Sciences, Changchun, China; 2 Shanghai Institute of Technical Physics, Chinese Academy of Sciences, Shanghai, China

**Keywords:** Micro-optics, Sub-wavelength optics

## Abstract

Professor Wei Lu is a leading scientist in infrared physics. He proposed the paradigm of localized manipulation over electrons and photons for infrared detection, addressing the critical challenge of dark current suppression in long-wave infrared detectors. His direct observation of the Haldane gap in quasi-one-dimensional magnetic materials was one of the earliest experimental validations of the Haldane’s conjecture - a crucial step in the theoretical discoveries of topological phases of matter that led to 2016 Nobel Prize in Physics for Duncan Haldane. Beyond fundamental research, Prof. Lu and his team developed a series of new advanced infrared detectors on multiple remote sensing satellite platforms. During his tenure as the Director of China’s State Key Laboratory of Infrared Physics and President of the Shanghai Institute of Technical Physics (SITP) at the Chinese Academy of Sciences, he led the strategic development of the institutions, contributing to China’s breakthroughs in spaceborne remote sensing technologies.


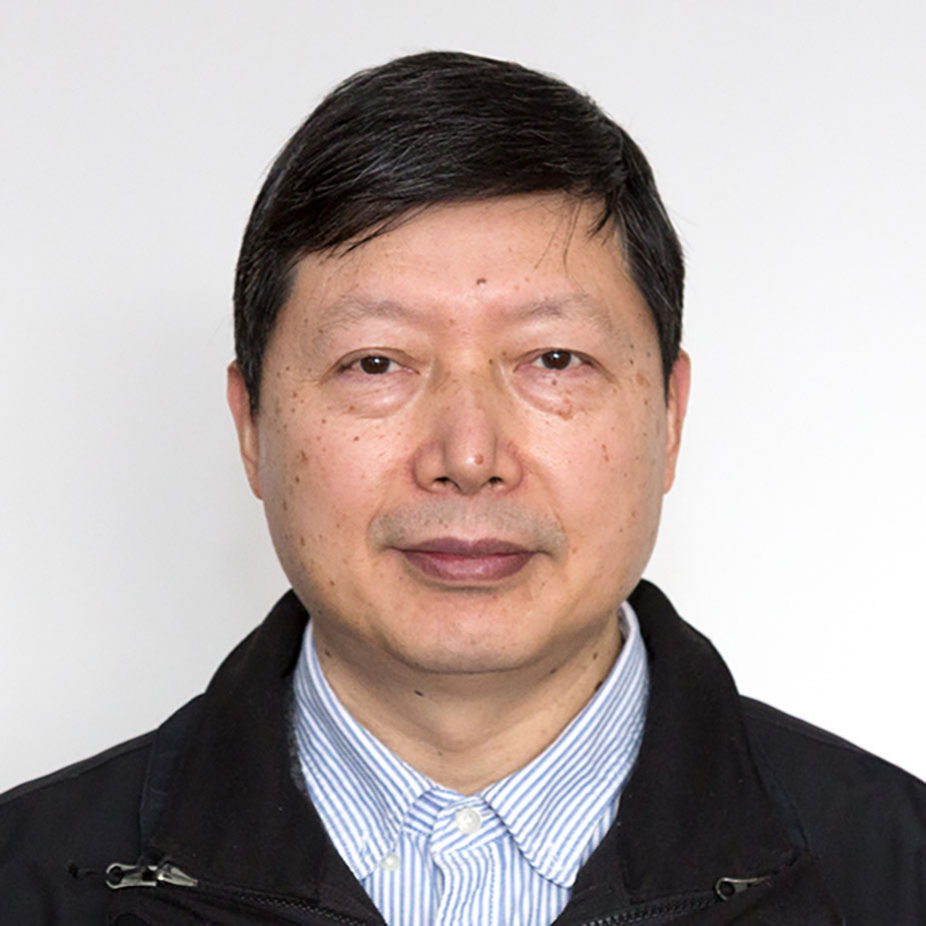


**Short Bio of Prof. Wei Lu**

Prof. Wei Lu received his bachelor degree from the Department of Physics, specializing in Lasers, at Fudan University in 1983. Then he received his Ph.D. from the Shanghai Institute of Technical Physics (SITP) of the Chinese Academy of Sciences (CAS) in 1988. From 1989 to 1991, he was an Alexander von Humboldt Fellow at the Technical University of Braunschweig in Germany. He has been working at SITP, CAS since 1988. From 1996 to 2000, he served as the Deputy Director of SITP. He was the Director of China's Key Laboratory of Infrared Physics from 2001 to 2011, and the Director of SITP from 2013 to 2018. From 2019 to 2025, he served as the Executive Dean of the School of Physical Science and Technology at ShanghaiTech University. He has long been engaged in the research of semiconductor optoelectronics science and technology. He was awarded the second prize of the National Natural Science Award of China in 2014 and the second prize of the National Technology Invention Award of China in 2011. He has published over 300 SCI papers and 3 monographs, with his work being cited over 15,000 times in SCI journals. He has also been granted more than 150 invention patents.

**Q1: Haldane’s conjecture is a crucial step in the theoretical discoveries of topological phases of matter that led to 2016 Nobel Prize in Physics for Duncan Haldane, and your direct observation of the Haldane gap in quasi-one-dimensional magnetic materials was among the earliest experimental evidence for the conjecture** ^1^ **. Could you share the story behind this discovery?**

A1: In 1980s, Duncan Haldane proposed ‘Haldane’s conjecture’ that an energy gap exists in the excitation spectrum of one-dimensional Heisenberg antiferromagnets with integer spins, while the half-integer spin chain is gapless, but direct experimental observation of the Haldane gap was lacking at that time. During my doctoral research – under the supervision by Prof. Xuechu Shen - I confirmed experimentally that, once extended phonons acquire quasi localized characteristics, the infrared absorption selection rule is generically lifted. Based on this idea, I had the feeling that there were chances we could verify ‘Haldane’s conjecture’. So in late 1980s, while working in Germany as a Humboldt Fellow, I collaborated with German and French colleagues and successfully observed an energy gap of a few meVs in a nickel-based quasi-one-dimensional antiferromagnetic material through far-infrared spectroscopy and high magnetic field experiments. This experiment was extremely challenging. Not only did we need to achieve ultra-low temperatures and strong magnetic fields, but we also had to overcome substantial noise interference during infrared measurements. When a distinct absorption peak appeared in the spectrum, I was very excited but remained cautious. After repeated verifications, we confirmed this gap was indeed the one predicted by Haldane. The results - published in 1991 - marked the first direct observation of the Haldane gap using infrared spectroscopy, providing pivotal evidence to support Haldane’s theory. Reflecting on that experience, I feel deeply honored to have contributed to this important chapter in condensed matter physics. This journey also reaffirmed my belief in the power of rigorous infrared spectroscopic experimentation to drive progress at the frontiers of theoretical physics.

**Q2: What motivated your research shift from infrared spectroscopy to infrared detection and its technologies?**

A2 : While investigating semiconductor quantum structures with in-situ modulated photoreflection spectroscopy, I directly observed quasibound states within a single quantum-step of AlGaAs-based heterostructures at room temperature (strong phonon scattering)^2^. These observations could not be explained by the conventional plane wave band model in the continuum approximation, so I proposed a localized-state theory for infrared detection, in which the formation of excited electronic states in quantum barriers is described through a localized-state framework. These findings along with their alignment with SITP’s long term research focus, marked a turning point of my research shift from infrared spectroscopy to infrared detection and technologies. At the same time, I have always deeply appreciated the crucial role of spectroscopic methodologies in exploring optical and physical research. Many physical phenomena cannot be directly observed, yet advanced spectroscopic measurements enable us to unveil microscopic physical processes, validate theoretical models, and even discover previously unknown mechanisms. These advanced experimental techniques powerfully support fundamental research findings, which in return, open new chances for devices and technologies.

**Q3: You mentioned the connections between fundamental research and device technologies. Could you elaborate what potential promise your work on nonequilibrium optoelectronic coupling and coherent control**^3,4^ **will hold for future device applications?**

A3: In 2018, we employed a scattering type scanning near-field noise microscopy to directly observe the nonequilibrium energy dissipation of electrons at the nanoscale^3^. We discovered that the effective peak temperature of electron does not align with the region of maximum current density and significantly exceeds the lattice temperature. Building on these insights, future developments may enable real-time thermal monitoring and modulation of localized heat, thereby improving the reliability of nanoelectronic devices. They may also open up possibilities for new types of infrared detectors based on hot-electron effects. In another recent study^4^, we introduced non-Hermitian exceptional points into photon-magnon coupled systems, significantly enhancing the performance of magnon frequency combs and enabling broadband output at low power levels. This new coherently controlled spin waves pave the way for on-chip broadband magnon frequency combs and innovative magnon-based devices, e.g., efficient on-chip radio frequence signal sources, broadband communication modules, novel high-sensitivity sensors integrating photonic and magnetic functionalities, etc.

Overall, we’re on the way to pushing the fundamental findings of nonequilibrium states and coherent phenomena into practical technologies and future devices.

**Q4: You and your team have developed optoelectronic silent states for ultrahigh light ellipticity discrimination** ^5^ **. With the sensitivity of infrared detectors approaching the intrinsic limit, what promise do you see in this work for potentially overcoming those limitations?**

A4: The detection scheme we propose in^5^ is a ‘differential’ approach in comparison with traditional ‘integral’ methods. Conventional infrared detectors collect signals from both the target and the background - an ‘integral’ process, whereas ours directly measures signal variations from the target. Specifically, we achieve this by precisely engineering two detection units with opposite optoelectronic responses. These units cancel each other out in the absence of a target signal, establishing what we call an ‘optoelectronic silent state’. In this silent state, the detector produces zero response to uniform background radiation, effectively suppressing background interference. Such silence will be broken even if a weak target signal emerges, allowing the detector to sensitively capture minor changes.

As infrared detectors approach their intrinsic sensitivity limit, thermal radiation from background - constrained by Planck’s law - often becomes the bottleneck to higher sensitivity. To overcome this, our ‘differential’ scheme suppresses the interference of background (i.e., suppress the dark current) and enables the detection of weak target signals. This holds great promise for applications requiring ultrahigh sensitivity in the presence of substantial background radiation, such as long-wave infrared Earth observation and deep-space exploration.

**Q5: A number of your developed devices have been implemented on satellites. Typically, the transition - from scientific discovery to functional devices, and ultimately to space applications - involves substantial challenges. How did you cope with them?**

A5: On one hand, I always closely align our fundamental research with the practical needs from aerospace. For example, as previously discussed, strong background radiation significantly degrades the infrared detection performance, and has become a crucial concern for aerospace infrared detectors. This challenge motivated us to explore the localized control theory, which further led to infrared detection mode achieving critical coupling. On the other hand, when developing new hyperspectral long-wave infrared detectors, we were determined not to stop at a mere proof-of-concept demonstration, instead we assembled interdisciplinary teams to systematically tackle practical challenges, including material growth, device design, and process optimization. As a result of aforementioned efforts, our detectors successfully integrated 46 long-wave infrared spectral channels on a single chip, compared to the previous best of four channels. Moreover, our detectors - implemented in satellites – demonstrated superior detection performance with efficient suppressing infrared thermal background radiation. By far, our devices have been implemented in 6 series of satellites including ‘Fengyun’. To summarize, I believe that if you have a clear sense of direction, remain committed to it, and consistently synergize ‘seeking fundamental breakthrough-resolving technological challenges-staying attuned to the practical needs of the aerospace applications’, you can carry scientific innovation all the way from the laboratory to orbit.

**Q6: You have previously served as Director of China’s State Key Laboratory of Infrared Physics and President of SITP, Chinese Academy of Sciences. As a leader in top-tier research institutions, how do you manage the institution’s research direction?**

A6: To guide the institution in the right direction, I think it’s essential to maintain a global perspective and a balanced strategic vision. During my tenure, I placed emphasis on aligning fundamental research closely with national priorities, so as to build up a complete innovation chain from fundamental physics to device development and ultimately to system-level applications. Specifically, on one hand, we constantly tracked cutting-edge developments across relevant disciplines, seeking to capture new mechanisms and technologies that could potentially reshape infrared physics. Following that strategy, we have intentionally brought in the research of metamaterials, two-dimensional materials, non-Hermitian physics, and artificial intelligence, and proactively integrated these areas into infrared optoelectronics research. On the other hand, by profoundly understanding national needs in the infrared physics domain - especially in the realm of space-based remote sensing technology - we set clear and application-driven research goals. Such goals should involve long-term consideration and frequent shifts in scientific goals should be avoided. Therefore, when setting such goals, we leveraged collective wisdom from a variety of experts. In addition, I appreciated openness and collaborations, spending efforts in attracting interdisciplinary talents to join our institutions. I believe that this balanced strategy - between fundamental innovation and practical applications - has enabled us to steer our research in the right direction.

**Q7: Over many years, your team has made pioneering innovations, spanning fundamental physics to aerospace applications. How do you cultivate talent to bridge innovation from fundamentals to applications?**

A7: Cultivating innovative talents bridging from fundamentals to applications requires providing them with various opportunities and well-structured mentoring. First, over the years, my team has maintained a balanced focus on both fundamental physics exploration and aerospace-application-driven research. Such diversity not only advances the team’s scientific goals but also offers invaluable training opportunities for young scientists, empowering them to both generate original ideas and push the boundaries of existing knowledge. Second, when collectively conducting projects, senior scientists pass down their knowledge and expertise to younger colleagues, establishing a well-structured mentoring. Third, we provide sufficient freedom for young researchers to pursue bold and innovative ideas. Under the above cultivation model, our team has gradually established innovation pathways linking fundamental research to practical applications, ensuring sustainable and forward-looking research development.

**Q8: In today’s landscape, how do you help stimulate the innovative potentials of young researchers?**

A8: First of all, providing young researchers with both intellectual freedom and stable, long-term support is essential. Intellectual freedom empowers them to propose original and potentially disruptive ideas, while alignment with practical needs ensures access to sufficient funding and experimental infrastructure. Equally important is encouraging them to be courageous in exploring new ideas - even those that may initially seem unconventional or impractical - as truly transformative breakthroughs often arise from radical thinking. It is also crucial to grant young researchers sufficient, uninterrupted time to focus on their scientific pursuits; I therefore strive to minimize their distractions from administrative responsibilities. Moreover, the atmosphere within the research group plays a vital role - young researchers should experience the excitement of innovation and the joy that comes from collaboration and open communication. To foster this, our senior researchers engage closely with younger colleagues to help them discover this excitement and joy - while carefully avoiding excessive intervention - ensuring they retain the space needed for independent intellectual growth. In short, I believe that creating an environment of trust, support, and understanding is essential for unlocking the creative potentials of young researchers.

**Q9: In your opinion, what directions might yield the next disruptive breakthroughs in the field of infrared optoelectronics?**

A9: I believe the next disruptive breakthroughs in infrared optoelectronics will likely emerge from two primary directions: advances in scientific understanding and technological capabilities. Both directions share the common goal of pushing the performance limit of infrared detectors. From the perspective of scientific understanding, the research paradigm for developing infrared detector chips is undergoing a significant shift: from traditional trial-and-error approaches to data-driven methodologies. As Prof. Herbert Kroemer famously said, the interface is the device. This transformation is particularly critical for advancing our understanding of interfaces - allowing us to move beyond the limitations of empirical knowledge and fundamentally reshape our comprehension of interfacial physics. Supported by China’s National Natural Science Foundation’s major instrumentation project, our team has recently evolved the traditional ‘structure-performance relationship’ at interfaces into a more precise ‘spectral-performance relationship’, utilizing high-throughput spectroscopy methods. By accurately probing localization phenomena of electronic states at the microscale, we’re able to translate empirical knowledge into a foundational scientific data platform, significantly accelerating infrared chip development through data-driven methodologies. From the other perspective of technological capabilities, infrared detection technologies are evolving from ‘detection’ towards ‘recognition’. One promising direction here is the integration of ‘recognition and computation’ into infrared detection chips, so as to enable a series of breakthrough: (a) advancing infrared detection from one-dimensional to multidimensional infrared information, which allows for more precise and reliable target recognition; (b) transitioning from conventional integral detection schemes toward differential detection methods, which enhances precise sensing of specific targets; (c) shifting from incoherent to coherent optoelectronic conversion, which effectively reduces detector noise and improves both sensitivity and accuracy. Both my team and many others have been exploring down this path, and we’re highly optimistic about the future potentials.

**Q10: You and your team have achieved metasurface-based photonic ruler and discovered the self-doping behavior** ^6,7^ **. How do you think these fundamental discoveries will potentially advance optical remote-sensing technologies?**

A10: We’ve just discussed about several promising directions in infrared optoelectronics, and the two works you mentioned show great promise in transforming optical remote-sensing technologies. In the metasurface-based photonic ruler work^6^, we use subwavelength metasurfaces to simultaneously capture multidimensional photon information such as frequency and polarization, decoding photons precisely just like a slide rule. This work suggests that spectral-polarimetric analyses - conventionally requiring bulky optical systems - could potentially be performed within a single chip. In the context of optical remote sensing, such possibility is very attractive in substantially reducing the satellite payload size and weight with significantly enhanced information gathering capability. Future devices, potentially as compact as a human hand, could efficiently perform hyperspectral and high-polarization remote sensing, providing unprecedented convenience for aerospace remote sensing. In the other work^7^, we discover that a variety of van der Waals materials are doping themselves from n- to p-type conductance with an increasing/decreasing layer-number. Taking advantage of this, we manage to construct homogeneous PN junctions in monolayer’s dimension/precision. These ultrathin junctions produce strong electric fields within extremely narrow dimensions, driving electrons into dissipationless transport regimes, thereby achieving near-ideal avalanche-like photomultiplication with high gain and low noise. This finding lays a robust foundation for designing highly sensitive infrared detectors that effectively suppress electronic scattering noise.

In summary, the above fundamental research has opened possibilities to on-chip spectral-polarization imaging and next-generation infrared focal-plane arrays, showing great potential to advance future optoelectronic devices and optical remote-sensing systems, equipping us with more precise and sophisticated observation capabilities.
